# Encapsulation of Alpha-Lipoic Acid in Functional Hybrid Liposomes: Promising Tool for the Reduction of Cisplatin-Induced Ototoxicity

**DOI:** 10.3390/ph15040394

**Published:** 2022-03-24

**Authors:** Manuela Curcio, Giuseppe Cirillo, Rosario Amato, Lorenzo Guidotti, Diana Amantea, Michele De Luca, Fiore Pasquale Nicoletta, Francesca Iemma, Mercedes Garcia-Gil

**Affiliations:** 1 Department of Pharmacy Health and Nutritional Science, University of Calabria, 87036 Rende, Italy; manuela.curcio@unical.it (M.C.); diana.amantea@unical.it (D.A.); michele.deluca@unical.it (M.D.L.); fiore.nicoletta@unical.it (F.P.N.); francesca.iemma@unical.it (F.I.); 2 Department of Biology, University of Pisa, 56127 Pisa, Italy; rosario.amato@biologia.unipi.it (R.A.); l.guidotti3@studenti.unipi.it (L.G.); mercedes.garcia@unipi.it (M.G.-G.); 3 Interdepartmental Research Center “Nutraceuticals and Food for Health”, University of Pisa, 56127 Pisa, Italy

**Keywords:** cisplatin-induced ototoxicity, lipoic acid, curcumin conjugate, functional nanoparticles, drug delivery, liposomes

## Abstract

In this study, in order to address the drawback of cisplatin (CDDP)-induced ototoxicity, we propose a straightforward strategy based on the delivery of a sulfur-based antioxidant, such as lipoic acid (LA), to HEI-OC1 cells. To this aim, hybrid liposomes (LA@PCGC) with a spherical shape and a mean diameter of 25 nm were obtained by direct sonication of LA, phosphatidylcholine and a gelatin-curcumin conjugate in a physiological buffer. LA@PCGC were found to be stable over time, were quickly (i.e., by 1 h) taken up by HEI-OC1 cells, and guaranteed strong retention of the bioactive molecule, since LA release was less than 20%, even after 100 h. Cell viability studies showed the efficiency of LA@PCGC for stabilizing the protective activity of LA. Curcumin residues within the functional liposomes were indeed able to maintain the biological activity of LA, significantly improving (up to 2.19-fold) the viability of HEI-OC1 cells treated with 5 μM CDDP. Finally, LA@PCGC was incorporated within an alginate-based injectable hydrogel carrier to create a formulation with physical chemical features suitable for potential ear applications.

## 1. Introduction

*Cis*-diamminedichloroplatinum(II), also known as Cisplatin (CDDP), is a cytotoxic drug widely used for the treatment of several types of solid tumours, including head and neck, lung, bladder, ovarian, and testicular cancers [1]. Similarly to many other conventional chemotherapeutics, CDDP exhibits nephrotoxicity, neurotoxicity, and ototoxicity [2,3,4], which, due to its progressive and irreversible nature, greatly affects the patient’s quality of life [5]. After entering cells, CDDP undergoes hydration and the loss of chlorine ions, leading to the cross-linking of nuclear and mitochondrial DNA [6,7], an increase in reactive oxygen species (ROS), and/or depletion of cell antioxidant defences [8], resulting in cell death due to the inhibition of DNA replication and cellular metabolism [9].

Different preclinical pharmacological strategies have been proposed to reduce the ototoxic effects of cisplatin, although none have been approved for clinical practice [10]. In addition to the use of nanoparticulate systems for increasing the therapeutic window of CDDP [11,12], data in the literature suggest that the administration of antioxidant agents such as N-acetyl cysteine, sodium thiosulfate, lipoic acid, as well as polyphenolic species (e.g., curcumin, rosmarinic acid, ferulic acid), can alleviate its toxic side-effects [13,14,15,16,17,18].

Lipoic acid (LA), an antioxidant compound soluble in lipids and water (in its reduced form, dihydrolipoic acid), is an essential cofactor for mitochondrial respiratory enzymes, and it is also widely distributed in either the cell membrane or intracellular compartments where it is involved in the aerobic metabolism [19,20,21]. Due to its ability to regenerate endogenous antioxidants (e.g., Vitamin C and E), and to act as a free radical scavenger and metal chelating agent [22], LA has been proposed for the treatment of different pathological conditions, including diabetes, neurodegeneration, and nephropathies [23,24,25]. More recently, LA was found to protect from CDDP-induced ototoxicity both in vitro and in vivo, and in cochlear explants (ex vivo) [3,26]. This protective effect was correlated with an increase in glutathione concentration, inhibition of lipid peroxidation, and the enhanced activity of antioxidant enzymes [26,27].

The low solubility of LA, together with its rapid degradation within the body, can be an obstacle for the development of effective LA-based therapeutic protocols, thus LA is often administrated in the form of an ethylenediamine complex or within nanoformulations, mainly based on phospholipids [28].

Although lipid nanoparticles have been perceived as ideal carriers for bioactive agents due to their similarity to cell membranes and high biocompatibility [29], their poor structural stability can result in degradation upon storage or a short half-life upon systemic administration [30,31]. To overcome these limitations, polymeric counterparts were added to lipid formulations to obtain hybrid nanosystems [32,33]. The ultimate aim of this approach was to enhance the therapeutic performance of the delivery vehicle by coupling the high biocompatibility and cell internalization efficiency of lipid structures with the high mechanical stability and chemical versatility of polymeric materials, which can be easily derivatized with many functionalities [34,35]. Among the different functionalization routes, it is well known that polymeric backbones can be modified by conjugation with antioxidant species, and that the resulting bioconjugates can be successfully used as starting materials for the development of functional carrier systems that are able to synergize the biological efficacy of the loaded therapeutic agents [36,37].

In this work, we encapsulated LA within a hybrid nanoformulation composed of phosphatidylcholine (PC) and a gelatin-curcumin conjugate (GC), and investigated its ability to counteract CDDP-induced cytotoxicity in HEI-OC1 cells, the most widely used cell line for investigating the biological responses associated with auditory cells and screening ototoxic drugs in vitro [38]. Moreover, to address the requirements for middle ear administration, such as increased residence time and accumulation through the round window membrane [39], the hybrid nanoparticles were embedded within alginate gels obtained by an ionic gelation process.

## 2. Results and Discussion

### 2.1. Synthesis and Characterization of Hybrid Liposomes

**Figure 1:**
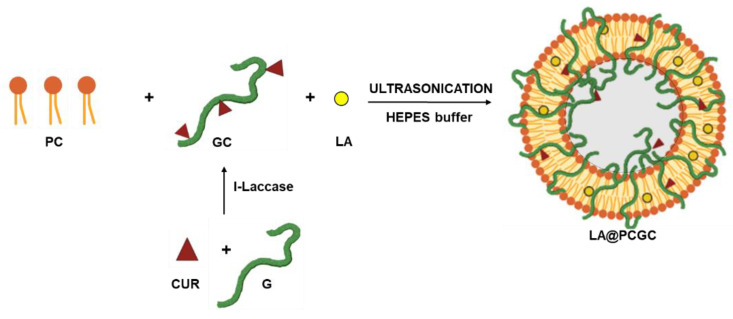
Schematic representation of LA@PCGC preparation. CUR, curcumin; G, gelatin; GC, gelatin-curcumin; I-laccase, immobilized laccase.

Hybrid liposome nanoparticles for the delivery of LA (bioactive element) to the inner ear, composed of PC and GC as the lipid and polymer counterparts, respectively, were prepared in a “one-step” method involving the ultrasonication of equal amounts of PC and GC in the presence of LA in an aqueous medium. This is a valuable alternative to traditional lipid film hydration methods, since it can be carried out in the absence of any trace of organic solvents [40], and it promotes the self-assembly of lipids into small spherical vesicles with a process that is more feasible to scale up (Figure 1).

The experimental procedure is composed of the following two-steps: (i) the enzymatic synthesis of the GC conjugate, and (ii) the sonication of the liposome components, that can be performed in a full green environment without the use of organic solvents to address the biocompatibility requirements of any pharmaceutical nanoformulation.

For the first step, enzyme coupling was selected for the synthesis of the GC conjugate (79 mg of curcumin per g) according to a previously developed protocol that exploits the eco-friendly advantages of the biocatalyst (immobilized laccase) and offers an easy way to recover the polymer bioconjugate (dialysis process) [41]. The reaction involves a Michael-type addition of nucleophilic groups to the side chain of the protein on an enzyme-activated polyphenol [42]. In detail, the laccase-induced oxidation of curcumin molecules to quinone derivatives is followed by the non-enzymatic attack of O, S, or, more predominantly, N nucleophiles [43]. The enzymatic independent step of the coupling is the driving force of the whole process that allows macromolecules of a different nature (e.g., proteins and polysaccharides) to be involved in the conjugation reactions with high degree of derivatization.

In a second step, since it has been proved that polymer-polyphenol conjugates can be used as functional components for highly engineered nanocarriers that are able to increase or even synergize with the therapeutic efficacy of the loaded drug [44], the GC conjugate was used as the polymer counterpart of a hybrid phospholipid nanosystem with the aim of enhancing the stability and therapeutic efficacy of the PC based-nanoparticles.

The rationale behind the choice of gelatin and curcumin for the synthesis of the bioconjugate was based on the peculiar properties of these two compounds in pharmaceutical formulations for ear applications. Gelatin is widely used for the preparation of drug delivery vehicles for the treatment of ear disorders such as vertigo, tinnitus, and hearing loss [45,46]. On the other hand, it has been proved that the administration of curcumin before and/or along with CDDP therapy exerts significant protective effects against CDDP-induced ototoxicity via multiple mechanisms related to its antioxidant properties, including anti-inflammation, restoration of a proper redox balance in the Corti organ, and enhancement of Nrf-2 translocation and heme oxygenase-1 in the cochlea [13,18]. Nevertheless, it is well known that the poor pharmacokinetic properties of curcumin dramatically limit its employment in clinical protocols [47,48], and that conjugation with macromolecular systems is an effective strategy for overcoming these drawbacks [49,50]. Moreover, we selected PC, a phospholipid constituting 45–60% of cell membranes, due to its well-known applicability to the fabrication of lipid-based nanosystems with high biocompatibility and cell uptake capability [51].

**Figure 2:**
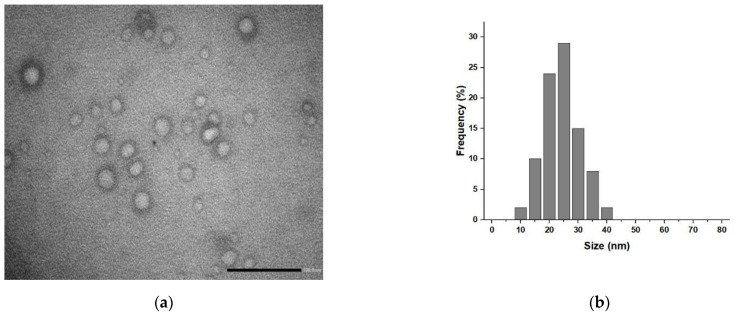
(**a**) TEM image of negatively stained LA@PCGC showing the spherical shape of the hybrid liposomes; (**b**) mean diameter distribution for LA@PCGC. Scale bar 100 nm.

For our formulation, transmission electron micrography (TEM) analyses (Figure 2) showed the presence of spherical vesicular systems, and the low-contrast interior of the nanoparticles indicated the presence of an aqueous core (Figure 2a) with a mean diameter distribution of 25 nm (Figure 2b).

**Figure 3:**
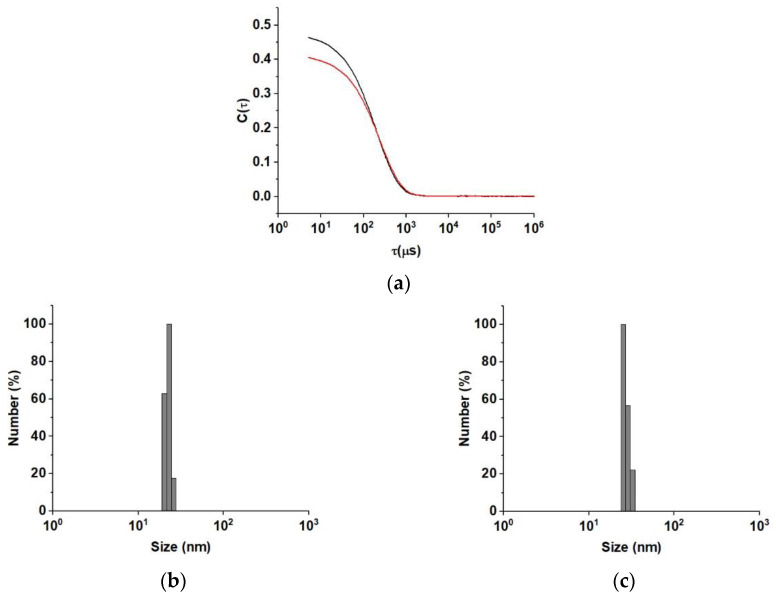

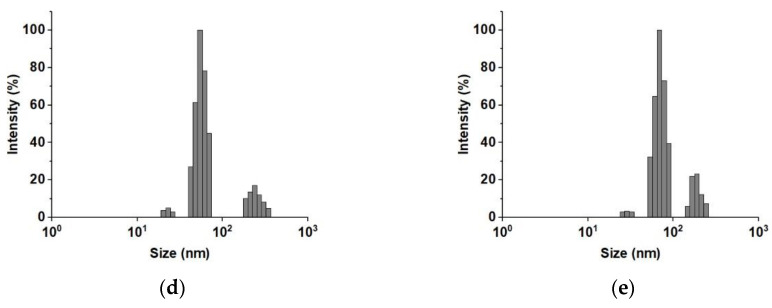
(**a**) DLS correlation functions, C(τ), as a function of correlation time, τ, for LA@PCGC at t = 0 (black line) and t = 1 month (red line) showing homogeneous liposome distribution and stability over time. (**b**,**c**) LA@PCGC number-weighted diameters at (**b**) t = 0 and (**c**) t = 1 month. (**d**,**e**) LA@PCGC intensity-weighted diameters at (**d**) t = 0 and (**e**) t = 1 month.

Moreover, dynamic light scattering (DLS) analyses (Figure 3) confirmed the quality and homogeneity of our liposome suspension (Figure 3a) with a polydispersity index (PDI) value of 0.223. The number-weighted (Figure 3b) and the intensity-weighted (Figure 3d) diameters were estimated as 22.85 and 54.17 nm, respectively, while a ζ-potential of +10.0 ± 0.9 mV was measured.

Interestingly, the formulation was found to be stable over time (Figure 3a), since no sign of aggregation was recorded, even after 1 month (PDI value of 0.293), confirming the high suitability of the nanoformulation as a carrier for a bioactive species. In detail, only slight modifications in the number-weighted (Figure 3d) and intensity-weighted (Figure 3e) diameters were recorded, with values of 25.19 and 68.84 nm, respectively, while the ζ-potential increased to +11.2 ± 1.1 mV.

### 2.2. Biological Characterization

Our biological characterization experiments were performed after exploring different LA to (PC + GC) ratios to find the optimal nanoparticle composition.

**Figure 4:**
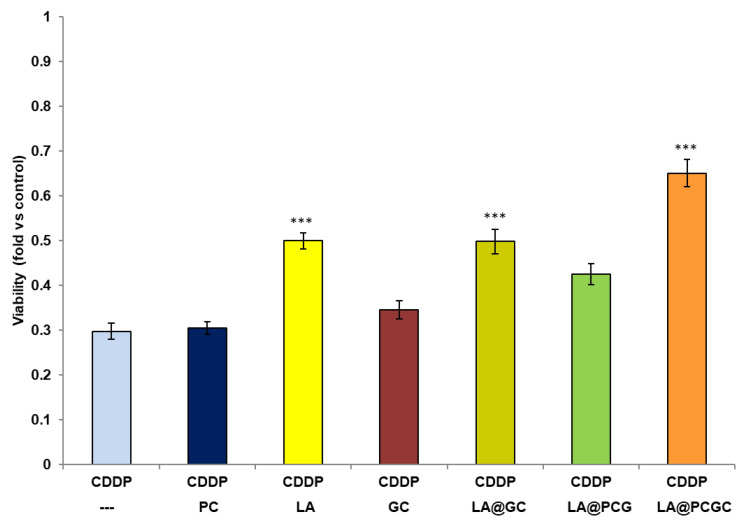
HEI-OC1 cell viability upon 48 h treatment in the presence of 5 μM CDDP and different combinations of 100 μg mL^−1^ PC, LA, GC, or gelatin (G). Viability was measured spectrophotometrically and normalized with respect to values obtained in the absence of CDDP. Data are plotted as the average ±SEM. The results were compared using a non-parametric ANOVA followed by Dunn’s multiple comparisons test. *** *p* < 0.001 vs. CDDP alone.

To determine the optimal concentration of PC for these experiments, its effect on HEI-OC1 cell viability was assessed over a concentration range 100–1000 μg mL^−1^ for 48 h. It was found that it was well tolerated up to 300 μg mL^−1^ (viability > 85%), but PC became toxic at higher concentrations, and cell viability was reduced to 43% at 1000 μg mL^−1^ (data not shown). Thus, 100 μg mL^−1^ was selected as the ideal PC concentration, since no sign of toxicity was recorded at this concentration in the presence of 5 μmol L^−1^ CDDP treatment (Figure 4).

As far as the amount of LA was concerned, in order to maximize the loading amount and guarantee a high intracellular level for effective cytoprotection, we selected a drug to carrier ratio of 50% corresponding to a LA concentration of 100 μg mL^−1^, which was within the active concentration range for the antioxidant, as suggested by the literature [21] for reaching an entrapment efficiency of 97%.

The efficiency of the LA@PCGC system acting as protective agent against CDDP-induced cytotoxicity of HEI-OC1 cells was assessed and compared to that of a control system prepared using native gelatin (G) instead GC (LA@PCG) (Figure 4).

It should be noted that, since PC was found to form an unstable formulation in the absence of protein-induced stabilization, the biological performance of the LA@PC system could not be tested.

As expected, a reduction in CDDP-induced toxicity was detected upon treatment with LA, with cell viability enhanced by almost 1.69-fold as a consequence of its strong antioxidant properties [19]. A slight increase in cell viability was recorded also in the presence of the GC conjugate (1.16-fold), although the effect was not significant at this concentration. Thus, it can be hypothesized that encapsulation within a functional hybrid liposomes formulation may increase the activity of bioactive LA.

The viability of HEI-OC1 cells exposed to CDDP in the presence of LA@PCGC was 2.18-fold higher than cisplatin alone, suggesting a possible synergistic effect between the encapsulated LA and CUR moieties of the liposomes. Notably, the combination of LA and GC was also found to be effective as a protective agent against CDDP toxicity, but the presence of PC within LA@PCGC was a key determining factor for guaranteeing an appropriate residence time for LA within the administration site.

**Figure 5:**
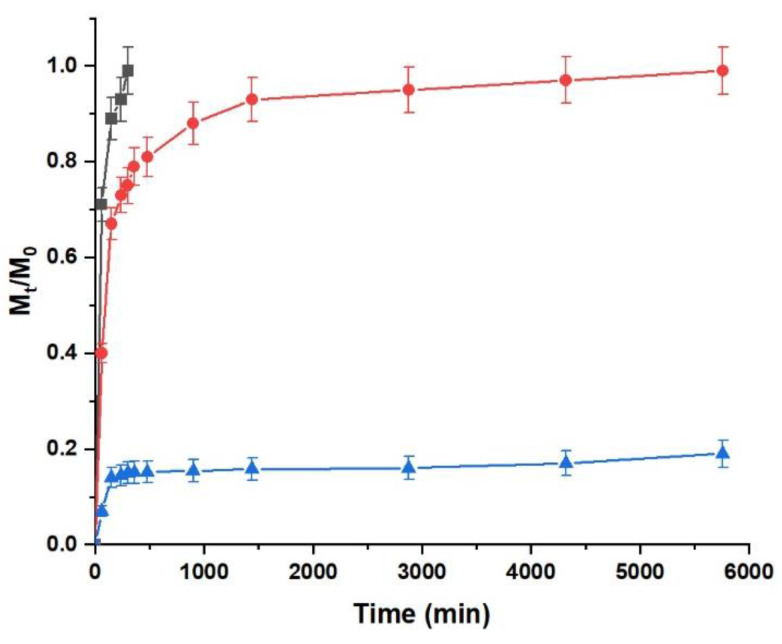
LA diffusion (grey curve) and the release profiles for LA@PCGC (blue curve) and LA@GC (red curve) in HEPES 10^−3^ M, pH 7.4. *M_t_*, amount of LA in solution at time t; *M*_0_, amount of loaded LA. Data are plotted as the average ± SEM.

The combination of LA and GC, although slowing the diffusion of LA due to the insurgence of weak interactions with the bioactive molecule and the modified protein [52], resulted in complete diffusion within 5 h (Figure 5). On the contrary, due to its high stability, the LA@PCGC formulation was found to strongly retain the loaded therapeutic agent, with the amount of released LA (*M_t_/M*_0_) being lower than 0.2, even after 96 h incubation.

The affinity of LA for the GC and PCGC systems was assessed by determining the kinetic parameters after applying an empirical model available in the literature [53], which considers the release of a bioactive molecule from a nanoparticle system as a partition between the carrier and the solvent phases according to the following first- and second-order kinetic equations (Equations (1) and (2)):

(1) $$\frac{M_{t}}{M_{0}}=F_{max}\left(1-e^{-\left(\frac{k_{R}}{F_{max}}\right)t}\right)$$

(2) $$\frac{M_{t}}{M_{0}}=\frac{F_{max}\left(e^{2\left(\frac{k_{R}}{\alpha }\right)t}-1\right)}{1-2F_{max}+e^{2\left(\frac{k_{R}}{\alpha }\right)t}}$$

where *F_max_* corresponds to the maximum amount of released drug (*M_t_*/*M*_0_) and *k_R_* is the release rate constant.

In this model, the *α* parameter is a measure of the physical chemical affinity of the drug for the carrier and the release media, and can be calculated as follows (Equation (3)):

(3) $$\alpha =\frac{F_{max}}{1-F_{max}}$$

Drug release occurs when *α* > 0.

The data reported in Table 1 clearly shows that a first-order model is a better fit for the experimental data (R^2^ > 0.95 in both cases).

From the analysis of the collected data, it is evident that the affinity of LA for PCGC is almost 57-fold higher than that for GC, confirming the importance of the stable vesicular structure of the hybrid liposomes formulation for the fabrication of an efficient delivery system. The calculated *k_R_* values also confirm the different release rates, although the increase (1.27-fold) is lower than that recorded for the α values, since *k_R_* is related to *F_max_* and not *M*_0_ in the applied model.

**Figure 6:**
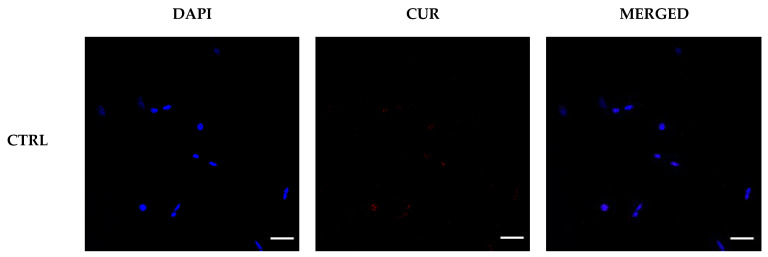

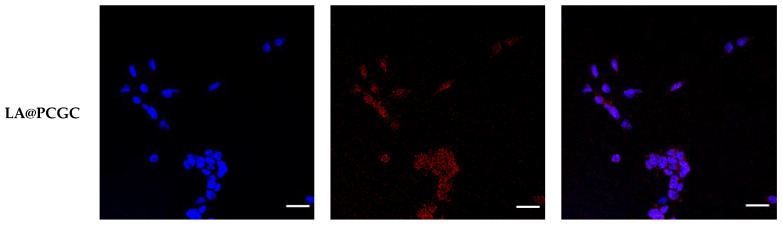
Confocal images of HEI-OC1 cells exposed to vehicle alone (CTRL) or treated with 100 μg mL^−1^ LA@PCGC for 1 h. The red signal corresponds to curcumin (CUR), whereas nuclei counterstained with DAPI are shown as a blue signal. Scale bar: 50 μm.

The effectiveness of LA vehiculation within HEI-OC1 cells with a negligible release of LA to the external media was confirmed by confocal microscopy analysis, where the presence of the fluorescence signal for curcumin was detected in the cytoplasm, even after 1 h incubation (Figure 6).

This finding is of great importance when considering the applicability of the proposed nanoformulation. Indeed, it is well known that in order to meet the requirements for prolonged inner ear delivery, nanoformulations should act as a drug reservoir, and their elimination by the Eustachian tube should be reduced [54]. The latter effect can be obtained by inclusion within an injectable hydrogel, which, due to non-thixotropic and viscoelastic properties, increases the residence time of the therapeutic agent in the middle ear [39].

Under our experimental conditions, we tested the possibility of incorporating LA@PCGC within an injectable hydrogel consisting of alginate (ALG) chains stabilized by ionic gelation in the presence of CaCl_2_ [55]. Preliminary viscoelastic measurements were performed to find either the appropriate LA@PCGC to ALG ratio or ALG crosslinking degree, taking into consideration the conditions needed to avoid perilymph leakage through the round window membrane, such as a physiological pH (~7.4) and a viscosity close to 5 (10^2^ Pa) [56]. We found that 0.5 mg mL^−1^ LA@PCGC in 0.90 mg mL^−1^ ALG, crosslinked in the presence of 0.12 mg mL^−1^ CaCl_2_, matched these requirements and could represent the final formulation to be employed in future ex vivo and in vivo experiments aimed at better elucidating the actual mechanism of action for the proposed system, as well as its in vivo applicability.

## 3. Materials and Methods

### 3.1. Hybrid Liposomes Preparation and Characterization

The hybrid liposomes formulation LA@PCGC was prepared with an ultrasonication method. Briefly, 12.5 mg PC, 12.5 mg GC, and 12.5 mg LA were added to 5 mL HEPES solution (10^−3^ M, pH 7.4) with stirring, then treated with a cup-horn high-intensity ultrasonic homogenizer (SONOPULS, Bandelin electronic GmbH, Berlin, Germany) equipped with a cylindrical tip (amplitude 70%, time 10 min). The same procedure was used to prepare LA@PCG when native G was used instead of the GC conjugate.

TEM images were recorded with a HRTEM/Tecnai F30 microscope and an accelerating voltage of 80 kV (FEI company, Hillsboro, OR, USA). For sample preparation, a drop of the liposomes dispersion was placed on a Cu TEM grid (200 mesh, Plano GmbH, Wetzlar, Germany), and the excess was removed with filter paper. Then, after deposition of a drop of phosphotungstic acid solution (2% *w*/*v*) on the carbon grid for 2 min, the sample was dried on air and analyzed.

The size distribution for the liposomes was determined at 25 °C by using a 90 Plus Particle Size Analyzer (Brookhaven Instruments Corporation, Holtsville, NY, USA) operating a 658 nm laser beam with the autocorrelation function set at 90°. The PDI was calculated from the instrumental data by using an inverse Laplace transformation and the Contin method. Homogenous and mono-disperse populations were characterized by PDI values ≤ 0.3 [57].

The ζ-potential of the hybrid liposomes formulation was determined at room temperature with laser Doppler electrophoretic mobility measurements using a Zeta-Sizer Nano ZS (Malvern Instruments Ltd., Malvern, UK). Values were calculated by the instrument software using the Helmholtz–Smoluchosky equation. Analyses were performed in triplicate and expressed as mean ± SEM.

### 3.2. Release Experiments

A volume of 2.0 mL LA@PCGC dispersion was loaded into a dialysis bag (MWCO 3.5 kDa) and dialyzed against 10.0 mL HEPES buffered solution (10^−3^ M, pH 7.4) at 37 °C with magnetic stirring. At suitable time intervals, 1.0 mL release medium was withdrawn, replaced with fresh medium, and the amount of released LA was quantified by UV-Vis analysis with an Evolution 201 spectrophotometer (ThermoFisher Scientific, Hillsboro, OR, USA) operating with 1.0 cm quartz cells at 320 nm.

From the calibration curves for LA under the same conditions, the cumulative amount of release was calculated using Equation (4):

(4) $$LA\;release\;\left(\%\right)=\frac{M_{t}}{M_{0}}\times 100$$

where *M_t_* and *M*_0_ are the amounts of LA in solution at time t and loaded into the liposome formulation, respectively. These experiments were performed under sink conditions.

All chemicals were obtained from Sigma/Merck (Darmstadt, Germany).

### 3.3. Cell Culture

HEI-OC1 (House Ear Institute-Organ of Corti 1) cells were maintained in high-glucose Dulbecco’s modified Eagle’s medium (DMEM) supplemented with 10% fetal bovine serum and 2.5 μg mL^−1^ amphotericin B at 33 °C in a humidified incubator with 10% CO_2_, as described [58].

PC, LA, GC, and their different combinations, were prepared immediately before use in DMEM without serum, sonicated, and diluted ten-fold to obtained the concentrations indicated in the experiments. G, PC, and LA were obtained from Sigma/Merck (Darmstadt, Germany); DMEM and fetal bovine serum were purchased from Euroclone (Milan, Italy).

### 3.4. Viability Experiments

Five thousand HEI-OC1 cells were seeded in 96-well plates containing 100 µL of medium. The following day, the cells were treated in the presence or absence of different concentrations of PC, LA, GC (and their combinations), and 5 μmol L^−1^ CCDD. After 48h, cell viability was measured with 3-(4,5-dimethylthiazol-2-yl)-2,5-diphenyltetrazolium bromide (MTT) reduction. Briefly, 0.5 mg mL^−1^ MTT in phosphate buffer saline was added to each well; cells were incubated at 33 °C in a humidified 10% CO_2_/90% air atmosphere for 60 min; the reaction was stopped by replacing the MTT solution with 100 µL dimethyl sulfoxide and the formazan salts were dissolved by gentle shaking for about 5 min at room temperature, and quantified spectrophotometrically by reading the absorbance at 596 nm with an automatic ultra-microplate reader, EL 808 (Bio-Tek Instruments Inc., Winooski, VT, USA).

The values were normalized against the average absorbance of the control sample. Each experiment was performed in octuplicate and repeated at least two times.

### 3.5. Confocal Microscopy Analysis

HEI-OC1 cells were seeded upon coverslips inside 6-well plates with a density of 1.0 × 10^5^ cells/well and cultured overnight in complete medium. Then, the cells were treated for 1 h with 100 µg mL^−1^ LA@PCGC. The cells were then fixed for 15 min at 25 °C using 4.0% paraformaldehyde and washed in PBS. A 0.2 µg mL^−1^ 4′,6-diamidino-2-phenylindole (DAPI) solution was employed to stain the nuclei. Slides were visualized (DAPI and curcumin excitation at 358 nm and 480 nm, respectively [59,60]) at 40× using a FV3000 confocal laser scanning microscope (Olympus Corporation, Tokyo, Japan).

### 3.6. Hydrogel Formulation

A solution of 0.5 mg mL^−1^ LA@PCGC was added to 0.90 mg mL^−1^ ALG in 5.0 mL HEPES buffered solution (10^−3^ M, pH 7.4) with magnetic stirring. Then, 0.12 mg mL^−1^ CaCl_2_ was added and the mixture was allowed to stand with stirring for 12 h. The rheometric measurements were carried out using a strain-controlled RFS III rheometer (Rheometric Scientific Inc., Piscataway, NJ, USA) equipped with plate-plate geometry (gap = 2 mm, Φ = 25 mm). The temperature was controlled using a Peltier system (25 ± 0.1 °C).

### 3.7. Statistical Analysis

Data are presented as the average ± SEM. The results of the viability assay were compared using a non-parametric ANOVA followed by Dunn’s multiple comparisons test. A value of *p* < 0.001 was considered significant.

## 4. Conclusions

In this work, we present experimental evidence that functional hybrid liposomes based on PC and GC conjugates represent a valuable strategy for delivering LA to HEI-OC1 cells and attenuating CDDP-induced cytotoxicity. The nanometric size of LA@PCGC ensured fast cellular uptake, while the high LA encapsulation efficiency, together with its slow-release profile, demonstrated the effectiveness of the proposed formulation as an intracellular delivery vehicle.

Our biological characterization clearly demonstrated the ability of the functional nanosystem to counteract CDDP toxicity in vitro, though future in vitro experiments will be required to better evaluate its therapeutic performance. To this end, preliminary formulation studies were performed by incorporating LA@PCGC within an injectable hydrogel and selecting a liposomes to hydrogel ratio matching the theoretical requirements for safe and effective ear administration. Future experiments will aim to evaluate the therapeutic applicability of the designed hybrid liposomes by determining the pharmacokinetics profile and the protective activity with a relevant in vivo model.

## Figures and Tables

**Table 1:** R^2^ values and kinetic parameters for Equations (1) and (2).

Mathematical Model	Parameter	LA	
		GC	PCGC
Equation (1)	R^2^	0.9571	0.9521
	*F_max_*	0.92	0.16
	α	10.88	0.19
	*k_R_* (10^−2^)	7.97	6.26
Equation (2)	R^2^	0.5625	0.4198

## Data Availability

Data is contained within the article.
